# ‘Triadic’ shared decision making in mental health: Experiences and expectations of service users, caregivers and clinicians in Germany

**DOI:** 10.1111/hex.13192

**Published:** 2021-01-15

**Authors:** Florian Schuster, Fabian Holzhüter, Stephan Heres, Johannes Hamann

**Affiliations:** ^1^ Klinikum rechts der Isar der Technischen Universität München: Klinik und Poliklinik für Psychiatrie und Psychotherapie München Germany; ^2^ kbo‐Isar‐Amper‐Klinikum München‐Ost, Klinik Nord München Germany

**Keywords:** caregiver, family involvement, mental health, quality of care, shared decision making, triadic decision making

## Abstract

**Background:**

Shared decision making (SDM) in mental health may contribute to greater patient satisfaction and is sometimes associated with better health outcomes. Here, SDM should not only involve service users and clinicians but also involve the service users' caregivers.

**Aim:**

This study aimed to achieve better insight into the current SDM patterns of triads of service users, caregivers and clinicians in inpatient mental health care and the three parties' expectations towards the prospects of triadic SDM.

**Design:**

The current research uses data from a representative cross‐sectional study on caregivers in psychiatric inpatient treatment. We analysed data on n = 94 triads of service users, their caregivers and their clinicians.

**Results:**

All three parties acknowledge caregivers to be of great support to monitor the progress with mental disease. The caregiver's role during consultations is most often described as being an expert, receiving or providing information and supporting service users. However, caregivers at times try to seek support for themselves during caregiver‐clinician interaction, or their behaviour was described as unhelpful. The potential prospects of caregiver involvement are clearly acknowledged despite the low implementation of caregiver involvement in this sample (only in one‐third of the cases).

**Conclusion:**

Triadic SDM rarely takes place in routine inpatient care. First, there should be a focus on interventions aiming at inviting caregivers to consultations. Only in the second step should a better conceptualisation of triadic SDM be undertaken.

**Public Contribution:**

Early results were discussed with a local peer support group for caregivers of individuals living with mental illness.

## 1 INTRODUCTION

Clinicians and patient representatives increasingly witness shared decision making (SDM) with service users experiencing mental health conditions as an ethical imperative,^1^ which may contribute to a greater service user satisfaction and is sometimes associated with better health outcomes (eg improved adherence to treatment and fewer relapses).^2^, ^3^ Shared decision making in general medicine^4^ and even more in mental health care should—on top of service users and clinicians—involve the service users' caregivers.^5^ In this regard, family and non‐family (eg friends and neighbours) caregivers are often referred to as ‘informal carers’ (also referred to as ‘caregivers’). Caregivers take on responsibility in areas that are insufficiently covered by health‐care professionals (eg monitoring medication and improving compliance)^6^, ^7^ and play a decisive role in coping with everyday life (eg finances, housing and social contact).^8^, ^9^ Consequently, caregivers are at a high risk of experiencing health, emotional and financial burdens themselves.^10^, ^11^ It is encouraging that caregiver involvement in psychiatric treatment does not only positively influence the service user's course of the illness but also improve the caregiver's health and well‐being.^12^, ^13^, ^14^, ^15^, ^16^ However, most SDM approaches do not explicitly involve the service user's caregivers.^17^ There are various reasons for this pattern, such as a lack of co‐operation between clinicians and caregivers in general^18^ and the worry that an inclusion of a third‐party may make SDM even more difficult.^17^ Moreover, there are insufficient data on how caregivers may be integrated into SDM. Therefore, the existing frameworks for the implementation of SDM (eg Ref. 19) do not yet offer concrete guidance for including third parties into SDM.

This study aimed to achieve better insight into the current SDM patterns of triads of service users, caregivers and clinicians in inpatient mental health care and the three parties' expectations towards the prospects of triadic SDM.

## 2 METHODS

Data of this study stem from a large, representative cross‐sectional study on caregivers in psychiatric inpatient treatment in psychiatric hospitals in Germany.^18^ In this study, n = 247 inpatients and their clinicians were interviewed. We also aim to address all service users' caregivers, which was possible in 94 cases. This study aimed to gain deeper insight into triadic SDM patterns of service users, caregivers and clinicians in inpatient mental health care and the three parties' expectations towards the prospects of triadic SDM.

### 2.1 Recruitment of participants and data acquisition

Data were collected for 10 months (October 2018 to August 2019). Recruitment took place on all wards of the participating hospital, except from wards with an emphasis on elderly psychiatry (65+ years) or alcohol/drug dependency. In each ward, clinicians were first asked for their next two to three service users pending discharge. These service users were then invited to be interviewed. Subsequently, all treating clinicians and, when possible, the service users' caregivers were interviewed. The caregivers were named by the service users and could be of any relation to the service users (informal carer). All structured interviews were performed face‐to‐face asking closed, semi‐open and open‐ended questions. In a first analysis of the data, service users and clinicians consistently reported that contact between the caregiver and the clinician in charge took place in only one‐third of the cases. The most important predictors for clinician‐caregiver contact included the service user's diagnosis (eg schizophrenia) and the treating hospital.^18^

We used data on n = 94 triads of service users, their caregivers and their clinicians for the present analysis. In particular, participants' answers to the following questions (displayed here is the service users' version of the questionnaire) were studied:

- ‘What role does your caregiver play, when it is about your illness?’
- ‘Has there been contact between your caregiver and the clinician in charge during your inpatient stay?’ (If yes) ‘What role did your caregiver play during this contact?’
- ‘Did you take action to make contact between your clinician and your caregiver happen?’
- ‘Were there any serious disagreements between your caregiver and your clinician during your inpatient stay?’
- ‘What is (or could be) the benefit of an involvement of your caregiver?’
- ‘What should this involvement of the caregiver ideally look like?’

All answers to these open‐ended questions were written down and later categorized to allow a descriptive analysis. For all participants, sociodemographic and clinical data were available.

### 2.2 Statistical analysis

In a first step, we inductively created categories based on the answers to the open‐ended questions. Two judges independently rated 30 answers as to whether these categories were present or not for each open question. Cohen's kappa coefficients (κ) ranged from 0.89 to 0.91 and thus indicated an excellent inter‐rater reliability.^20^ In the second step, these categories were then coded for all participants, and descriptive statistics was performed via SPSS Statistics 25 (eg frequencies, mean values).

### 2.3 Ethics

An ethical and legal review of the study was done by the local ethics committee. The ethics committee raised no objections to the conduct of the study.

## 3 RESULTS

### 3.1 Participants

We included 94 triads of service users, caregivers and clinicians in this study. The original sample of 247 service users and the subgroup of 94 service users comprised in the triads did not differ substantially in age, gender, diagnosis and severity of illness. Table 1 shows the sociodemographic data of service users.

**TABLE 1:** Characteristics of service user sample (n = 94)

Variable	Frequency (n, %)	Range, mean, SD
Age		19‐84, M = 43.8, SD = 17.0
Gender		
Female	57, 60.6%	
Male	37, 39.4%	
Main diagnosis		
Affective disorder	58, 61.7%	
Schizophrenia or delusional disorder	25, 26.6%	
Personality and behavioural disorder	5, 5.3%	
Psychic and behavioural disorder through psychotropic substances	3, 3.2%	
Neurotic disorder	2, 2.1%	
Organic psychic disorder	1, 1.1%	
CGI		M = 4.4, SD = 1.2
GAF		M = 51.3, SD = 15.9

Abbreviations: CGI, Clinical Global Impression; GAF, Global Assessment of Functioning.

The 94 caregivers included in this analysis were aged between 21 and 90 (M = 53, SD = 15.4) and predominantly female (66.0%). Thirty‐two caregivers were parents (34.0%), followed by 23 partners (24.5%), 14 siblings (14.9%), six children (6.4%) and three aunts (3.2%). Only a minority of caregivers were not a family member (13 caregivers, 13.8%), and three were categorized as ‘others’ (3.2%).

### 3.2 What role do caregivers play in the context of psychiatric disorders?

**FIGURE 1:**
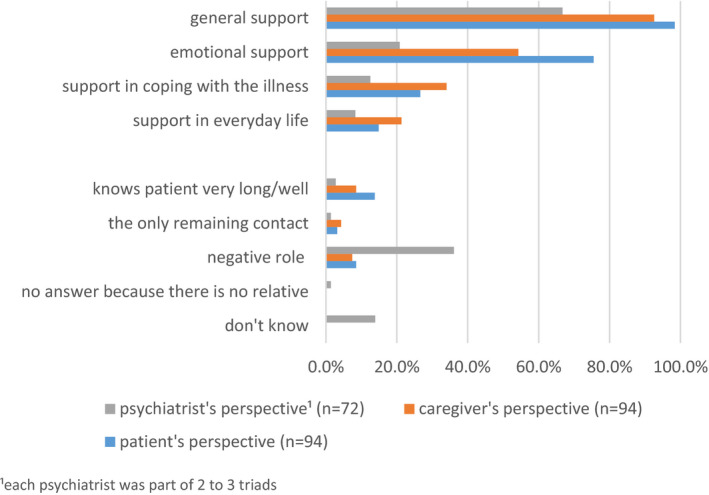
Role of the caregiver in regard to the service user's illness

The most often cited category here was ‘support’, which was reported by service users, caregivers and clinicians in 98.4%, 92.6% and 66.7% of the cases, respectively. This category was further subdivided into ‘emotional support’, ‘support in everyday life’ and ‘support in coping with the illness’. Service users, caregivers and, to a lesser extent, clinicians stated that ‘emotional support’ is the most relevant aspect of support in general. However, clinicians attributed a negative or detrimental role to 36% of the caregivers. They described the caregivers' influence as either causing or sustaining the mental illness, or perceive the caregiver to be mentally ill themselves. An overview of the different roles caregivers played according to the participants' replies is provided in Figure 1.

### 3.3 What role do caregivers play during service user‐caregiver‐clinician contact?

**FIGURE 2:**
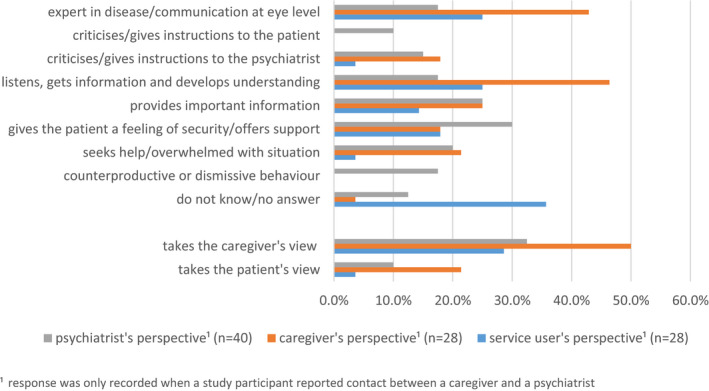
Role of the caregiver during service user‐caregiver‐clinician contact

Many service users could not provide an answer (35.7%) regarding the caregivers' role during service user‐caregiver‐clinician communication. The most frequently reported answer from the service users' view though was that caregivers were seen as experts in the disease (25.0%) and seeking information (25.0%). Caregivers themselves most often reported that they wanted to acquire information (46.4%), followed by the self‐perception as an expert in the disease (42.9%). The clinicians' perspective differed substantially. They most frequently described the caregivers' role as giving the service user a feeling of security and offering support (30.0%), followed by the caregiver providing important information (25.0%). Clinicians mentioned inappropriate behaviour of the caregiver in 17.5% of the cases (Figure 2).

Two more sets of categories were derived from the participants' answers. First, we categorized whether the caregivers primarily wanted to express their own opinion during caregiver‐clinician interaction or if they wanted to make the service user's voice heard. Here, all three parties more often reported that the caregivers wanted to express their own opinion rather than the service user's opinion (28.6% service users, 50.0% caregivers and 32.5% clinicians). Second, we assessed how frequently caregivers were seeking help for themselves during meetings with clinicians. This topic was mentioned by clinicians and caregivers in approximately 20% of the cases (Figure 2).

Overall, only 15 service users (16.0%), 38 caregivers (40.4%) and 26 clinicians (27.7%) had actively tried to initiate caregiver‐clinician interaction.

### 3.4 Conflicts between caregivers and clinicians

In total, 33 service users (35.1%), 30 caregivers (31.9%) and 22 clinicians (23.4%) reported about conflicts being related to inpatient treatment. Most participants described conflicts between service users and clinicians. Only one service user (1.1%), nine caregivers (9.6%) and two clinicians (2.1%) reported conflicts between caregivers and clinicians. This service user reported that the family did not understand why he is back in the hospital again; in their opinion, no treatment was needed. Conflicts often arose from different perceptions of what the cause of the illness is, leading to different expectations regarding treatment according to perspective of the caregivers. Furthermore, caregivers felt that the clinicians did not take them seriously and found it difficult to establish contact with them. Finally, hospital regulations (eg time of admission, exit rules and time of discharge) led to further conflicts. The two clinicians complained that the caregivers had no understanding of the mental illness and gave the service users too little time to recover.

### 3.5 What is (or could be) the benefit of an involvement of the caregiver?

**FIGURE 3:**
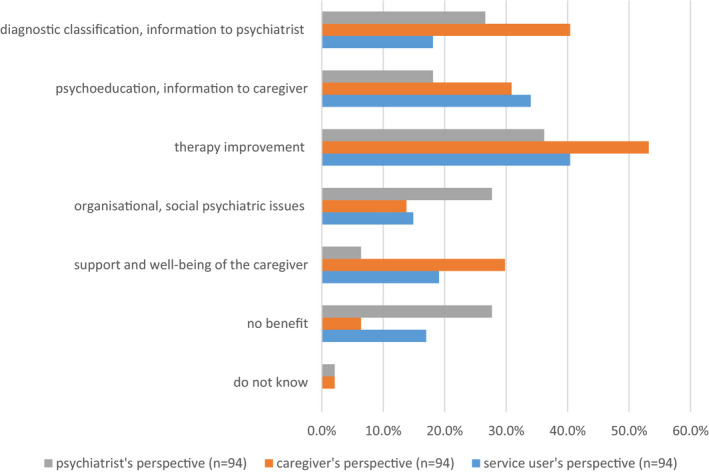
Benefit of caregiver involvement

All three parties (service users, caregivers and clinicians) most often stated that caregiver involvement is beneficial and improves therapy (40.4%, 53.2% and 36.2%). Many service users (34.0%) think clinicians can use caregiver‐clinician interaction to provide information to the caregiver regarding mental illness. Moreover, caregivers more often see contact with the clinician as an opportunity to contribute important information to the treatment (40.4%). From the clinicians' perspective, caregiver involvement is often perceived as a chance to clarify organizational and social issues (27.7%) closely followed by acquiring information from the caregiver (26.6%). Furthermore, 17.0% of service users and 27.7% of clinicians think caregiver involvement is not beneficial at all. An overview of the anticipated benefit of caregiver involvement from the perspectives of the three parties surveyed is provided in Figure 3.

### 3.6 What should involvement of the caregiver ideally look like?

**FIGURE 4:**
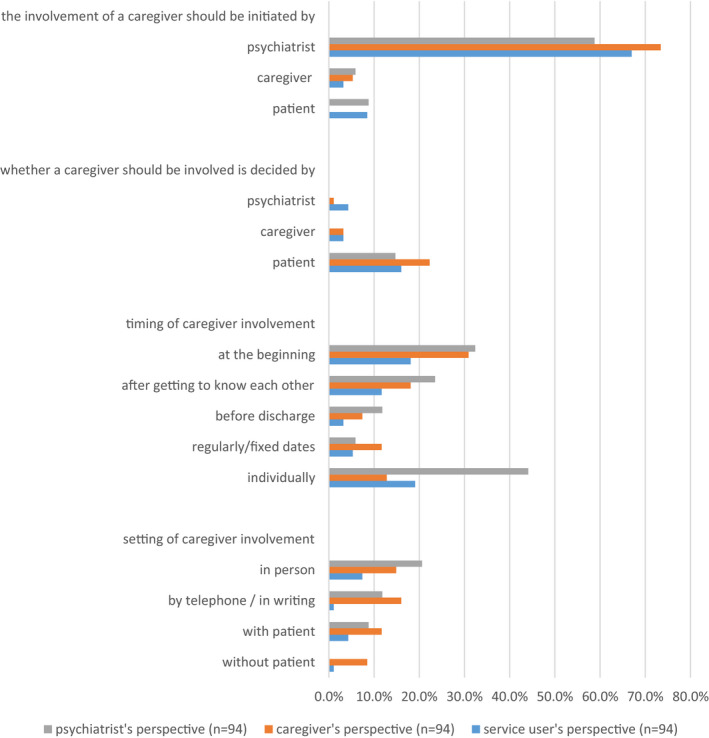
Expectations of the ideal caregiver involvement

Although respondents gave a wide range of answers, some major categories regarding initiation, extent, timing and setting of involvement could be identified. The majority of interviewees (67.0% of service users, 73.4% of caregivers and 58.8% of clinicians) supported the idea that the initiative for caregiver involvement should come from the clinician in charge of the inpatient treatment. Thus, all three parties (16.0% of service users, 22.3% of caregivers and 14.1% of clinicians) mention that the service user should ultimately decide whether or not there will be caregiver‐clinician interaction. As for the ideal timing of caregiver involvement, the majority of service users (19.1%) and clinicians (44.1%) fancy an individualized approached contrasted by 30.9% of caregivers who favour their own involvement at the beginning. Service users (7.4%) and clinicians (20.6%) report accordingly most often that the caregiver should be involved in person if it comes to the ideal setting of caregiver involvement. Interestingly, caregivers (14.9%) most frequently wish for caregiver‐clinician interaction through telephone or writing. Figure 4 shows the details concerning expectations towards the ideal caregiver involvement.

## 4 DISCUSSION

Caregivers are viewed as an important part in supporting their relatives dealing with mental illness. Their role is often described as being an expert, receiving or providing information and supporting service users during consultations with clinicians, which seldom take place. However, caregivers were sometimes seen as seeking support themselves or as acting unhelpfully. The potential prospects of caregiver involvement are clearly acknowledged despite the low implementation of caregiver involvement in this sample. Some guidance as to how involvement could best take place is given.

### 4.1 Strengths and limitations

This investigation is one of the few looking into service user‐caregiver‐clinician triads. It is important to highlight that all interviews were performed face‐to‐face using questionnaires alongside open‐ended questions. We could gain a deeper insight into the caregivers' role in matters of SDM by choosing this approach.

We ended up with 94 triads (out of 247 service user cases) in our study despite our best efforts. It is conceivable that the caregivers interviewed represent a subgroup having better relationships with service users or showing more interest in the service users' course of disease than average. We had to limit our selection to the 94 cases in which a complete triadic data set was available (service users, caregivers and clinicians) because we wanted to explore the role of the caregiver from the perspectives of all three parties involved. This approach could lead to biased results.

Participants generally had difficulties to answer our questions or remained rather vague with their answers during the investigation. This is probably due to the fact that many participants have not given sufficient thought about the caregivers' role in matters of SDM or are strongly influenced by the existing hospital routines.

Another limitation is that we were not able to observe conversations between service users, caregivers and clinicians but only received reports about them.

### 4.2 The findings in the context of shared decision making

What do our findings tell us about the actual implementation of triadic SDM? The results are quite clear regarding the current state of the implementation of triadic SDM. For the majority of inpatients, triadic SDM does not happen, simply because caregivers and clinicians do not interact at all. For the remaining 30% for which service user‐caregiver‐clinician contact had been established, it can only be estimated from our results to what extent SDM had taken place during these meetings. There may be some 20%‐40% of caregivers that are seen as minimally helpful or as concerned with their own burden. For these, it is questionable whether or not triadic SDM had happened. For the remaining, the quotes from participants allow us to suppose that at least some steps of SDM were undertaken. Thus, the important components of SDM are being an expert, supporting the service user, making the service user's view heard and providing information.

How can our findings contribute to the further development of a triadic SDM model? In a recent publication, Hamann and Heres^17^ proposed the three‐talk model for the implementation of SDM by Elwyn et al^19^ as a possible basis for the development of a triadic SDM approach. The three‐talk model consists of three recursive steps. In the first step (team talk), service users and clinicians need to clarify who else should join the team for decision making and what is the caregiver's role in a triadic SDM approach according to Hamann and Heres. In the following step (option talk), the available options should be discussed, and in the final step (decision talk), a decision should be made based on preferences. In our opinion, the findings of the current study can be attributed best to the first phase of the three‐talk model. Here, it should be discussed who is involved in the process of SDM. All three parties (service users, caregivers and clinicians) agree that the initiative to involve a caregiver must come from the clinician at this stage according to our investigation. This is especially important as other research shows that caregivers want to participate in SDM and typically feel excluded.^21^ Hamann and Heres^17^ further proposed that the caregiver's role should be clarified in the first phase. Our investigation shows that caregivers not only want to acquire information but also want to be perceived as an expert on mental disease. Service users who were able to answer this question also mentioned these two categories most frequently. Furthermore, the first phase of the three‐talk model should be used to clarify whether the caregiver wants to be involved in person, by telephone or via the Internet. Quotes from participants did not significantly contribute information to phases 2 (option talk) and 3 (decision talk) of the three‐talk model, so no new conclusions for them could be drawn.

Further issues attributable to SDM were cited, including an improvement of therapy (eg by providing additional information) or helping to implement therapeutic decision into the service user's daily life only when asked about the prospects of triadic SDM. These factors may be linked to ‘option talk’ (eg adding important information and there by adding options) or ‘decision talk’ (eg which option actually could be implemented).

### 4.3 Implications for clinical practice

Participants' suggestions of what involvement of caregivers should look like are rather vague and do not give clear cut information on how caregivers may best be integrated into SDM. In our opinion, this might be due to the fact that all three parties (service users, caregivers and clinicians) have not yet given sufficient thought to the inclusion of caregivers in triadic decision making. Thus, the focus should be on the initiation of meetings between service users, caregivers and clinicians before triadic SDM can be better conceptualized, especially for the steps of option talk and decision talk. From our point of view, the most important finding here is that the clinicians are expected to initiate these meetings, which is also in line with existing recommendations.^22^ In addition, to allow more caregivers to participate, clinicians might consider alternatives to face‐to‐face meetings. In our investigation, caregivers stated that they wish to communicate with the clinician in charge through telephone or writing. It is essential to think about new ways of service user‐caregiver‐clinician communication to improve and facilitate the involvement of caregivers in the process of SDM. A recent review of online interventions for families of service users with severe mental disorders shows that online interventions are both well‐accepted and beneficial for service users and caregivers.^23^

There are other areas in medicine where the involvement of caregivers in decision making is more widespread than in mental health care (eg oncology), and consequently, the conceptualization of triadic SDM is more advanced.^24^, ^25^, ^26^ Laidsaar‐Powell et al used this precondition in oncology to develop a guideline on how to involve caregivers and how to deal with challenging interactions (TRIO Guidelines‐1 and TRIO Guidelines‐2). Therefore, it is essential to consider interventions promoting caregiver involvement in psychiatry (eg Refs 16, 27). With the aim to encourage caregiver involvement and recovery, Dixon et al^16^ tested a manualized protocol using principles of SDM. In a two‐step approach, service users and clinicians first worked on individualized recovery goals and clarified whether or not a caregiver should be involved to achieve these goals. They were invited to the second step, if service users wished their caregivers to be involved. Dixon et al^16^ were able to show that this standardized procedure leads to more family participation and better service user outcome. In 2019, Kaselionyte et al demonstrated that a structured approach may facilitate service user‐caregiver‐clinician interaction. In their intervention, the clinician did not have to be a psychiatrist but could belong to another group of professionals (eg social therapists and nurses). Kaselionyte et al discussed the possibility of caregiver involvement directly after hospital admission and, if the service user consented, arranged a meeting within the first week of inpatient treatment. However, the authors argue that it is hard to implement such a new approach without additional staff support. Furthermore, as part of an intervention, it should be considered which member of the multi‐professional team (eg nurses, psychologists or social workers) could be involved more in the treatment if the involvement of a caregiver is not possible or further support is needed.

## 5 CONCLUSION

It was the aim of the current investigation to gain a deeper insight into SDM patterns of triads of service users, caregivers and clinicians in inpatient mental health care and the three parties' expectations towards the prospects of triadic SDM. It is hardly possible to derive a concrete concept of triadic SDM from there because the participants' answers remained rather vague and the contact between service user, caregiver and clinician rarely took place. In our view, the first step should be to focus on interventions that aim at inviting caregivers to consultations and only in the second step should a better conceptualization of triadic SDM in mental health be undertaken.

## CONFLICT OF INTEREST

Dr Hamann reports grants from Janssen‐Cilag, Germany, during the conduct of the study, and personal fees from Janssen‐Cilag, Germany, Otsuka and Lundbeck, outside the submitted work. Dr Heres reports grants from Janssen‐Cilag, Germany, during the conduct of the study, and personal fees from Janssen‐Cilag, Germany, Otsuka and Lundbeck, outside the submitted work. Dr Holzhüter and Mr Schuster have nothing to disclose.

## Data Availability

The data that support the findings of this study are available on request from the corresponding author. The data are not publicly available due to privacy or ethical restrictions.
